# Transcriptome analysis of the brain provides insights into the regulatory mechanism for *Coilia nasus* migration

**DOI:** 10.1186/s12864-020-06816-3

**Published:** 2020-06-18

**Authors:** Meiyao Wang, Gangchun Xu, Yongkai Tang, Pao Xu

**Affiliations:** 1 Key Laboratory of Freshwater Fisheries and Germplasm Resources Utilization, Ministry of Agriculture, Freshwater Fisheries Research Center, Chinese Academy of Fishery Sciences, Wuxi, 214081 China; 2 Wuxi Fisheries College, Nanjing Agricultural University, Wuxi, 214081 China; 3 Aquatic Animal Genome Center of Freshwater Fisheries Research Center, Chinese Academy of Fishery Sciences, Wuxi, 214128 China

**Keywords:** *Coilia nasus*, Brain, Transcriptome, Salinity, Stress

## Abstract

**Background:**

*Coilia nasus* (*C. nasus*) is an important anadromous fish species that resides in the Yangtze River of China, and has high ecological and economical value. However, wild resources have suffered from a serious reduction in population, attributed to the over-construction of water conservancy projects, overfishing, and environmental pollution. The Ministry of Agriculture and Rural Affairs of the People’s Republic of China has issued a notice banning the commercial fishing of wild *C. nasus* in the Yangtze River. Wild *C. nasus* populations urgently need to recover. A better understanding of *C. nasus* migration patterns is necessary to maximize the efficiency of conservation efforts. Juvenile *C. nasus* experience a simultaneous effect of increasing salinity and cold stress during seaward migration, and the brain plays a comprehensive regulatory role during this process. Therefore, to explore the early seaward migration regulation mechanism of juvenile *C. nasus*, we performed a comparative transcriptome analysis on the brain of juvenile *C. nasus* under salinity and cold stress simultaneously.

**Results:**

Relevant neurotransmitters, receptors, and regulatory proteins from three categories of regulatory pathway play synergistic regulatory roles during the migration process: neuronal signaling, the sensory system, and environmental adaptation. The significant differential expression of growth-related hormones, thyroid receptors, haptoglobin, and prolactin receptors was similar to the results of relevant research on salmonids and steelhead trout.

**Conclusions:**

This study revealed a regulatory network that the brain of juvenile *C. nasus* constructs during migration, thereby providing basic knowledge on further studies could build on. This study also revealed key regulatory genes similar to salmonids and steelhead trout, thus, this study will lay a theoretical foundation for further study on migration regulation mechanism of anadromous fish species.

## Background

The *Coilia* fish belongs to the family of Engraulidae and the order of Clupeiforme, and is distributed in the mid-west Pacific and Indian oceans. As a popular *Coilia* fish species for consumers in China, *Coilia nasus* (*C. nasus*) is a precious fish species in the Yangtze River. It is one of the “Three Delicious Species in the Yangze River”, with Reeve’s shad (*Tenualosa reevesii*) and obscure pufferfish (*Takifugu fasciatus*) being the other two species [1, 2]. However, it has suffered from a serious population reduction in recent years as a result of the over-construction of water conservancy projects, overfishing, and environmental pollution [3–5]. Consequently, the catch yield has reduced by 60% and continues to drop yearly [6]. It has been included on the “National Key Protective Species List” of China. The Ministry of Agriculture and Rural Affairs of the People’s Republic of China has issued a notice banning the fishing of wild *C. nasus* in the Yangtze River for production. The restoration of wild *C. nasus* is urgently needed.

*C. nasus* is an important anadromous fish species. In February, mature adults return to their native Yangtze River and its tributaries to spawn. Their offspring move to the estuaries, where they will remain until autumn, and then migrate to the ocean for growth and fattening [7, 8]. Therefore, during this process, juvenile *C. nasus* is simultaneously exposed to increased salinity and cold stress. There has been very few research on regulation mechanism of *C. nasus* during migration, which were mainly on regulatory pathways and function of key regulatory genes that function during spawning migration, such as the comparative transcriptome analysis on brain and liver of wild adult *C. nasus* during spawning migration [9] and function analysis on FoxL2 and Cyp19a1of *C. nasus* during anadromous migration [10]. The results indicated that many neurotransmitter signaling pathways in brain and relevant receptors, transporters, and regulatory proteins were significantly upregulated. Meanwhile, most pathways in liver were downregulated and indicated its function in energy conservation during spawning migration. The brain serves as the center of the nervous system in vertebrates and exerts a more comprehensive regulatory function than other tissues of perception system regulation, learning, and memory muscle activity, through which the organism responds to the changing environment [11, 12]. Therefore, research on the influence of environmental factor variation on the brain transcriptome will be beneficial for revealing the comprehensive regulatory network that is formed during *C. nasus* migration.

Traditionally, research on the effects of temperature and salinity as environmental stressors in fish has been carried out in the liver and gills due to the pivotal roles of these organs in energy supply and osmoregulation. Recent studies that investigated the strengthening of the brain regulatory function in response to salinity and cold stress have indicated that the expression of hormones, neurotransmitters, receptors, and key regulatory proteins was upregulated [13–18]. Xu et al. [19] investigated the effect of cold exposure on the brain transcriptome of the Yellow rum (*Nibea albiflora*). The results indicated that the most significantly enriched pathway was involved in signal transduction. Salmonids, such as Atlantic salmon (*Salmo salar*), coho salmon (*Oncorhynchus kisutch*), and steelhead trout (*Oncorhynchus mykiss gairdneri*), in addition to *C. nasus*, are also economically important anadromous fish species. In order to explore their regulatory mechanisms during smoltification, some research has been carried on trout, and resident and migratory salmonids, including comparative transcriptome analyses of the brain, liver, gill, kidney, and olfactory rosettes [20–24]. The results of these analyses indicated that differentially expressed genes (DEGs) were mainly involved in development and metabolism [20, 21]. Relevant research on Atlantic salmon indicated that DEGs were involved in electron transport, oxygen transport and endocrinology, there was no change in the expression of thyroid-stimulating hormone (TSH), which is different from the results of similar research on steelhead trout and coho salmon [20, 22–24]. Additionally, a comparative transcriptome analysis on coho salmon in freshwater and early marine environments showed that differential regulatory pathways in the brain were mainly involved in protein synthesis and MHC1-mediated antigen presentation [24]. These studies indicated that anadromous fish species have differential regulatory mechanisms during seaward migration. Therefore, it is essential to explore the regulatory patterns in different anadromous fish species to reveal the potential universal regulatory mechanisms.

Research on the regulatory mechanism of *C. nasus* during migration is still in its infancy. Juvenile *C. nasus* seaward migration is an important part of the species’ life history, but relevant research has not been carried out. Given the simultaneous effects of salinity and cold stress that juvenile *C. nasus* experiences during seaward migration, we performed a comparative transcriptome analysis of the brain under saline and cold stress, to investigate the regulatory role that the brain of juvenile *C. nasus* plays during migration. We aimed to reveal key regulatory pathways and genes, in order to construct a regulatory network; lay the theoretical foundations for further research on regulatory mechanisms during *C. nasus* migration and for the optimization of artificial breeding of *C. nasus*, which is beneficial for providing high-quality fry fish for proliferation and release; and contribute to efforts towards the restoration of wild *C. nasus*. This study will also lay a theoretical foundation for research on the regulation patterns of global *Coilia* fish during migration. Combined with existing reports on anadromous fish, this study will collect basic information on the regulation mechanism of anadromous fish species during migration.

## Results

To comprehensively explore regulation mechanism of juvenile *C. nasus* during seaward migration, we performed comparative transcriptome analysis on juvenile *C. nasus* under saline and cold stress simultaneously. Top 10 GO terms, top 10 KEGG pathways and key DEGs were obtained after library construction, sequencing, data filtering, assembly, annotation and differential expression analysis. Correlation analysis on intraclass difference in the control and stressed group was made, validation of RNA-Seq data was carried out with quantitative real-time polymerase chain reaction (qPCR).

### Transcriptome assembly and statistics of unigenes

The average RIN (RNA Integrity Number) for six brain samples was 9.5. After quality filtering, the RNA-Seq of six brain samples yielded around 46.36 million high-quality sequence data. The Q value (Q30) was used as the cutoff for quality control. The Q30 values of the samples reached up to 93.03%, and the GC-content of each sample reached around 48.5% (Table 1). The clean reads obtained from the six transcriptome libraries were assembled to full-length transcripts, and a total of 436,325 unigenes were obtained after the elimination of redundant transcripts. The average transcript length was 795 bp, and N50 was 1001 bp. The average clean ratio reached 99.8%.

**Table 1:** Statistics of sequencing reads

Samples	C1^a^	C2^a^	C3^a^	S1^a^	S2^a^	S3^a^
Raw reads	46,644,438	46,486,085	46,608,652	46,401,430	46,355,046	46,667,900
RIN	9.3	9.1	9.6	9.7	9.7	9.4
Clean reads	46,544,340	46,390,980	46,502,440	46,328,580	46,266,780	46,570,300
Q30	92.43%	91.61%	92.28%	92.70%	92.59%	92.31%
(G + C) content	47.00%	47.50%	47.00%	47.00%	48.00%	47.50%
Clean ratio^b^	99.79%	99.8%	99.79%	99.84%	99.81%	99.8%

^a^C1-C3 refers to three replicated groups of the control group, S1-S3 refers to three replicated groups of the stressed group

^b^Clean ratio equals clean reads/raw reads

### Correlation analysis on intraclass differences in the control and stressed group

CORREL function was used to analyze difference of FPKM (Fragments per kilobase of transcript per million mapped reads) of DEGs in the three replicated groups of control group (C1-C3), as well as in the stressed group (E1-E3) (Additional file 5: Table S4). The correlation analysis results of C1-C2, C2-C3, C1-C3, E1-E2, E2-E3 and E1-E3 were as follows, y = 0.835x + 0.9861and R^2^ = 0.8554 (correlation coefficient r = 0.924863193), y = 1.1849x - 1.2712 and R^2^ = 0.9373 (r = 0.968150821), y = 1.0599x - 0.7987 and R^2^ = 0.92 (r = 0.959142331), y = 0.8144x + 1.0789 and R^2^ = 0.7603 (r=,0.969855973), y = 0.9511x + 0.9828 and R^2^ = 0.9081 (r = 0.935047937), y = 0.9119x + 0.204 and R^2^ = 0.889 (r = 0.937179862). The results indicated that replicated groups in the control group had strong correlation, as well as in the stressed group, intraclass difference were both small in these two groups. These differences were mainly caused by the individual differences of experimental animals and operation difference during experiment, which are normal and acceptable difference. Therefore, Intra-group differences did not affect the further analysis on differences between the control and stressed group.

### Top 10 gene ontology (GO) enrichment analysis on DEGs

**Fig. 1:**
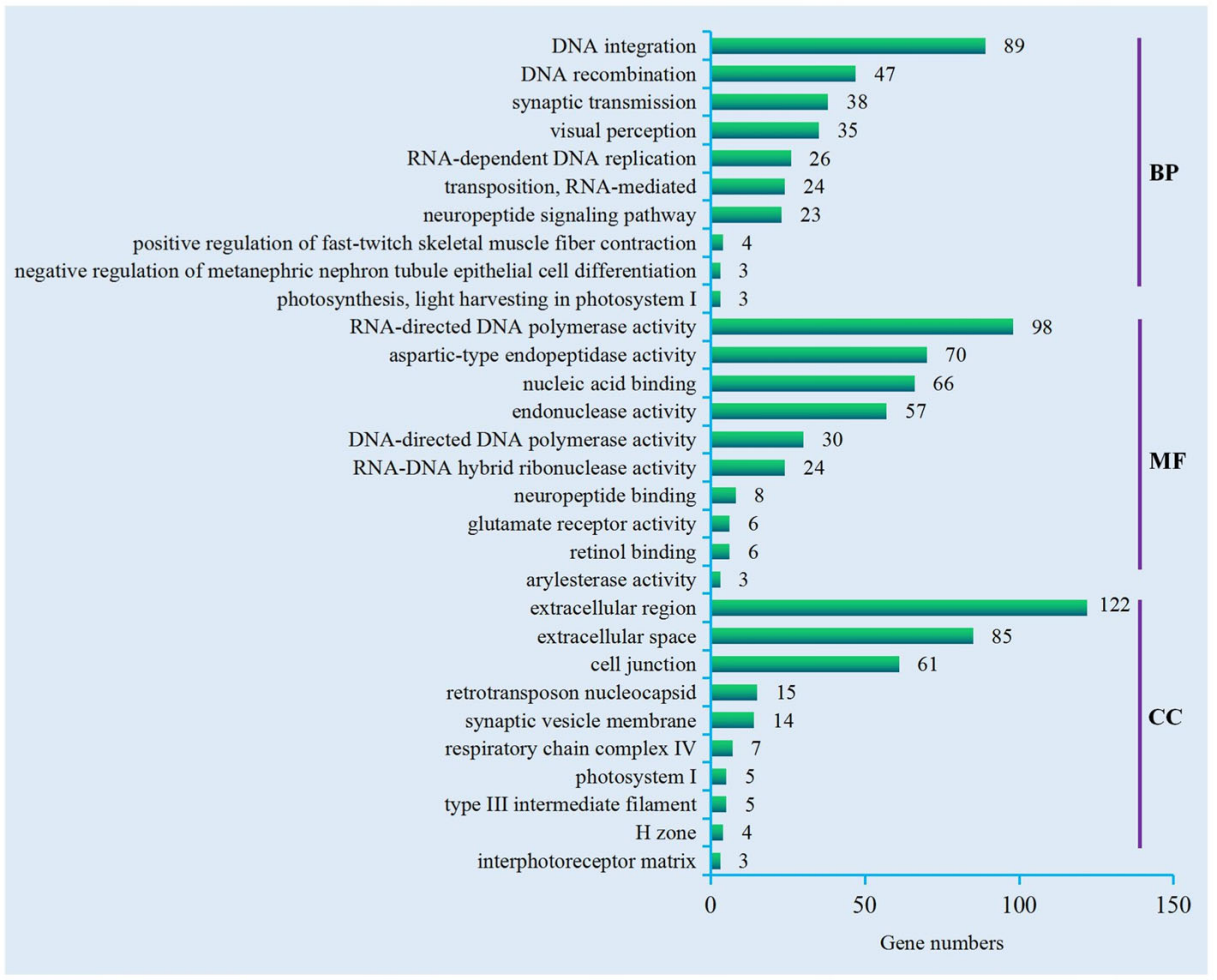
Top10 GO terms. Top 10 GO terms were enriched from DEGs of the *C. nasus* brain transcriptome. Number of DEGs enriched in each term is shown at the right side of the bar. The vertical bar shows the three categories that the GO terms were enriched in: BP, MF, and CC

Based on the GO enrichment analysis, 38,579 unigenes were categorized into 62 functional groups from three categories: biological processes (BP), molecular functions (MF), and cellular components (CC) (Additional file 1: Figure S1). Then, we conducted a further GO enrichment analysis on DEGs and obtained the top 10 GO terms from each of the three categories (Fig. 1). Most BP terms, with the exception of some involved in the general function (Nos. 1, 2, 5, and 6), were related to neuronal signal transduction (Nos. 3 and 7) or the sensory system (Nos. 4 and 10). Most MF and CC terms were relevant to synaptic transmission or the sensory system and relevant components, such as neuropeptide binding, glutamate receptor activity, the synaptic vesicle membrane, the cell junction, retinol binding, photosystem I, the interphotoreceptor matrix, etc. The DEGs of each term are shown in Additional file 2: Table S1.

### Top 10 Kyoto encyclopedia of genes and genomes (KEGG) enrichment analysis

**Fig. 2:**
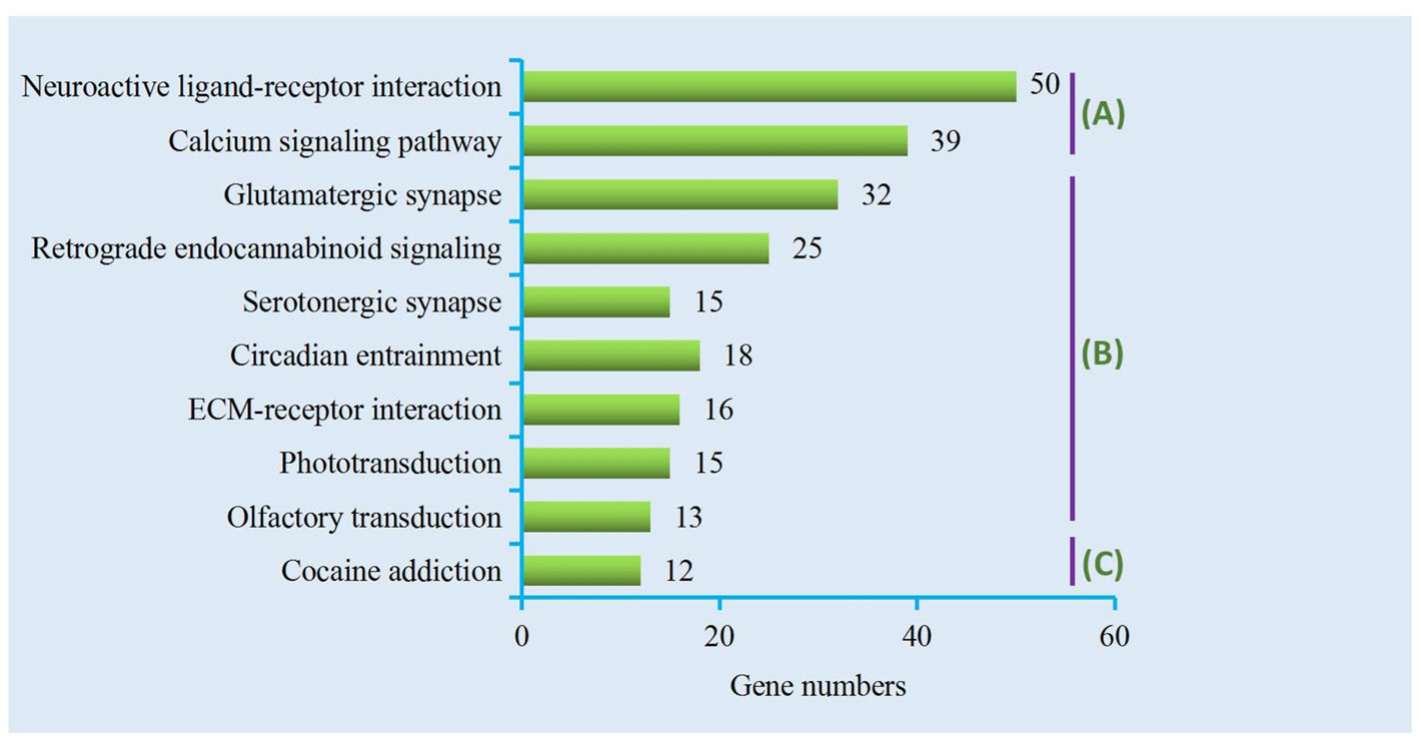
Top10 KEGG pathways. Top 10 KEGG pathways were enriched from DEGs of the *C. nasus* brain transcriptome. Three capital letters indicate three main categories: (**a**), Environmental Information Processing; (**b**), Organismal systems; (**c**) Human Diseases

In total, we obtained 4721 DEGs (Additional file 2: Table S1); 2020 DEGs were downregulated and 2701 DEGs were upregulated. As shown in Fig. 2, five pathways were involved in neuronal signaling, including neuroactive ligand-receptor interaction, the calcium signaling pathway, glutamatergic synapse, retrograde endocannabinoid signaling, and the serotonergic synapse. Two pathways were related to the sensory system—olfactory transduction and phototransduction—and two were relevant to environmental adaptation—circadian entrainment and ECM–receptor interaction. The DEGs involved in these pathways are shown in Additional file 2: Table S1.

### Functional analysis on DEGs

According to a pathway hierarchy (Additional file 3: Table S2), the top 10 GO and top 10 KEGG terms indicated that the DEGs were mainly involved in three categories: neuronal signaling, the sensory system, and environmental adaptation. The relevant DEGs are listed in Table 2.

**Table 2:** Differentially expressed genes in response to salinity and cold stress

Category	Gene name	Gene definition	Log_**2**_FoLdchange	***P***-value	Up/Down ^**a**^(+/-)	
**Signal transduction**	ADCY2	adenylate cyclase 2	1.623024029	1.69565E-06	+	
	AHNAK	Neuroblast differentiation-associated protein	1.23991252	1.8059E-05	+	
	CBLN1	Cerebellin-1	1.477382614	2.64918E-05	+	
	CARTPT	Cocaine- and amphetamine-regulated transcript protein	1.705475308	2.57562E-05	+	
	EPHA4	Eph receptor A4	2.789583201	1.35975E-06	+	
	GRIN2A	glutamate receptor ionotropic, NMDA 2A	1.857980995	3.70302E-05	+	
	GRIA1	glutamate receptor 1	1.688055994	3.12289E-05	+	
	GRM5	metabotropic glutamate receptor 5	2.364836686	3.71795E-06	+	
	GABRD	gmma-aminobutyric acid receptor subunit gamma	5.95631015	9.03684E-16	+	
	GAL	Galanin peptide	27.63200829	4.90E-12	+	
	GALR1/R2	galanin receptor 1	2.606657572	4.26707E-05	+	
	GLDN	Gliomedin	2.294447358	5.23949E-06	+	
	GNG2	guanine nucleotide-binding protein G (I)/G (S)/G (O) subunit gamma-2	4.727920455	5.78933E-06	+	
	GNGT1	guanine nucleotide-binding protein G (T) subunit gamma-T1	7.078259014	6.39E-10	+	
	HTR4	5-hydroxytryptamine receptor 4	3.754887502	1.50417E-05	+	
	LRRTM4	Leucine-rich repeat transmembrane neuronal protein 4	2.331514144	1.57678E-05	+	
	MCH2	Pro-MCH 2	25.97596269	2.10002E-07	+	
	NCS1	Neuronal calcium sensor 1	2.047305715	1.22204E-05	+	
	NPBWR2	neuropeptides B/W receptor 2	4.054765803	2.71068E-09	+	
	NPY	neuropeptide Y	1.627628966	2.82109E-06	+	
	NSG1	Neuron-specific protein family member 1	1.047174058	4.29656E-05	+	
	NTSR1	Neurotensin receptor type 1	3.137503524	1.05811E-05	+	
	OPRL1	nociceptin receptor	1.521397372	3.59681E-05	+	
	PENKB	proenkephalin B (prodynorphin)	3.242856524	1.6675E-07	+	
	PNOC	Prepronociceptin	2.532269607	4.4328E-07	+	
	RIMS	regulating synaptic membrane exocytosis protein	1.002266607	2.38297E-05	+	
	SIPA1L1	signal-induced proliferation-associated 1 like protein 1	2.019469864	5.7446E-06	+	
	SV2	MFS transporter, VNT family, synaptic vesicle glycoprotein 2	1.105773138	3.32163E-05	+	
	SLC18A1_2	MFS transporter, DHA1 family, solute carrier family 18 (vesicular amine transporter), member 1/2	5.129283017	1.5945E-05	+	
	SLC1A	solute carrier family 1 (glutamate transporter), member 7	4.169925001	2.75989E-05	+	
	SLC6A1	solute carrier family 6 (neurotransmitter transporter, GABA) member 1	-1.795641501	3.94984E-05		–
	SNAP25	synaptosomal-associated protein 25	5.087462841	1.77515E-05	+	
	STAT	signal transducer and activator of transcription	2.359895945	4.93096E-05	+	
	SYNPR	Synaptoporin	5.087462841	5.35768E-08	+	
	SYT1/10	synaptotagmin-1	1.503662399	2.83789E-05	+	
	TAAR	trace amine associated receptor	3.5698751	2.98658E-05	+	
	TAC1	tachykinin 1	2.510961919	3.78253E-06	+	
	TENM1	Teneurin-1	2.895530733	8.37792E-07	+	
	OPRL1	Nociceptin receptor	1.521397372	3.59681E-05	+	
**Sensory system**	AIPL1	Aryl-hydrocarbon-interacting protein-like 1	6.087462841	1.07977E-08	+	
	ANO7	Anoctamin -7	1.179072643	1.89574E-05	+	
	AVPR1B	Vasopressin V1b receptor	24.45513053	2.58472E-05	+	
	CLDN	Claudin-22	3.896164189	6.32318E-07	+	
	CNGA2/CNGB1	cyclic nucleotide gated channel alpha 2/beta 1	17.93156857	1.96876E-05	+	
	CRYAB	crystallin, alpha B	1.201986211	1.9046E-05	+	
	EYA4	Eyes absent homolog 4	-2.411813598	1.34683E-05		–
	GABRB	gamma-aminobutyric acid receptor subunit beta	3.005805622	6.20322E-07	+	
	GNAT1_2	guanine nucleotide-binding protein G (t) subunit alpha 1/2	-4.727920455	5.78933E-06		–
	GPRC5C	G-protein coupled receptor family C group 5 member C	25.21697079	3.47461E-06	+	
	GUCA1	guanylate cyclase activator 1	5.673002535	9.35629E-07	+	
	LRAT	phosphatidylcholine-retinol O-acyltransferase	25.95393638	2.31133E-07	+	
	LXN	Latexin	-1.459431619	5.90012E-06		–
	NCKX1	solute carrier family 24 (sodium/potassium/calcium exchanger), member 1	3.554588852	2.60147E-05		–
	PDE1	calcium/calmodulin-dependent 3’,5’-cyclic nucleotide phosphodiesterase	1.58282359	1.02849E-05	+	
	PDE6A/6B	rod cGMP-specific 3’,5’-cyclic phosphodiesterase subunit alpha/beta	-5.882643049	1.50714E-06		–
	RCVRN	recoverin	-1.734266445	1.66801E-05		–
	RGR	RPE-retinal G protein-coupled receptor	3.022367813	2.50182E-05	+	
	RHO	rhodopsin	-5.736965594	1.46417E-11		–
	RLBP1	Retinaldehyde-binding protein 1	-3.594181031	6.71346E-07		–
	RP1L1	Retinitis pigmentosa 1-like 1 protein	1.834221528	1.9372E-05	+	
	RPE65	retinoid isomerohydrolase	2.777607579	4.71976E-07	+	
	STRC	Stereocilin	-3.222392421	2.61719E-05		–
	TAS1R1	taste receptor type 1 member 1	-3.544320516	4.83161E-05		–
	TBR1	T-box brain protein 1	30.21465692	3.06E-28	+	
	TECTA	Alpha-tectorin	-2.725283789	2.72437E-06		–
	TMC2	Transmembrane channel-like protein 2	3.896164189	6.32318E-07	+	
	TRPC3	Short transient receptor potential channel 3	-4.85077616	5.48984E-26		–
	VAX1	Ventral anterior homeobox 1	25.21697079	3.47461E-06	+	
**Stress response**	AHCY	adenosylhomocysteinase	-3.689214537	1.72215E-05		–
	AMD1	S-adenosylmethionine decarboxylase	-2.136372442	3.90066E-06		–
	AQP9	aquaporin-4	4.882643049	3.44583E-06	+	
	ATP1B	sodium/potassium-transporting ATPase subunit beta	-1.026216857	1.43307E-05		–
	CLDN	claudin	3.896164189	6.32318E-07	+	
	CYP51	sterol 14-demethylase	4.24879339	4.57294E-05	+	
	GHRH	Somatoliberin	4.307428525	9.87095E-07	+	
	HP	Haptoglobin	2.797137522	6.13977E-08	+	
	HSP70	Heat shock protein 70	1.985378817	6.00422E-08	+	
	METE	5-methyltetrahydropteroyltriglutamate--homocysteine methyltransferase	5.459431619	1.97921E-06	+	
	METK	S-adenosylmethionine synthetase	4.64385619	1.96876E-05	+	
	MTHFD	methylenetetrahydrofolate dehydrogenase	1.949534933	1.07109E-05	+	
	NTS	Neurotensin	5.544320516	9.11866E-11	+	
	PRLR	prolactin receptor	1.870147682	2.42871E-05	+	
	SLC6A5	Sodium- and chloride-dependent glycine transporter 2	3.440566897	1.10089E-06	+	
	SSTR1	somatostatin receptor 1	4.247927513	2.91451E-06	+	
	TACR3	tachykinin receptor 3	-2.473931188	1.14954E-05	+	
	TRPC3	Short transient receptor potential channel 3	-5.48984E-26	1.9283E-24		–
	RGS9	regulator of G-protein signaling 9	1.205267382	2.78147E-05	+	
	PRDX1/6	peroxiredoxin 6, 1-Cys peroxiredoxin	24.93156857	7.99857E-06	+	
	ATP1A/1B	sodium/potassium-transporting ATPase subunit alpha/beta	5.977279923	2.8038E-07	+	
	CACNB4/CACNG4	voltage-dependent calcium channel beta-4/gamma-4	1.086335169	4.49908E-05	+	
	CADPS2	Calcium-dependent secretion activator 2	3.736965594	3.10145E-05	+	
	CORIN	Atrial natriuretic peptide-converting enzyme	1.595769256	2.17862E-05	+	
	GRIK1	glutamate receptor, ionotropic kainate 1	1.857980995	3.70302E-05	+	
	KCNA10	Potassium voltage-gated channel subfamily A member 10	2.475733431	1.21502E-06	+	
	KCNE1	potassium voltage-gated channel Isk-related subfamily E member 1	4.794415866	2.64187E-07	+	
	KCNIP1	Kv channel-interacting protein 1	1.478302393	3.18614E-05	+	
	KCNJ3	potassium inwardly-rectifying channel subfamily J member 3	1.538699778	1.97736E-05	+	
	KCNQ1	potassium voltage-gated channel KQT-like subfamily member 1	3.938599142	3.938599142	+	
	SCN4AB	Sodium channel protein type 4 subunit alpha B	4.266786541	6.43397E-12	+	

^a^Up/Down: DEGs upregulated or downregulated compared to the control group

Upregulated DEGs were those with log2Foldchange > 0, and downregulated DEGs were those with log2Foldchange < 0

### Validation of RNA-Seq data by qPCR

**Fig. 3:**
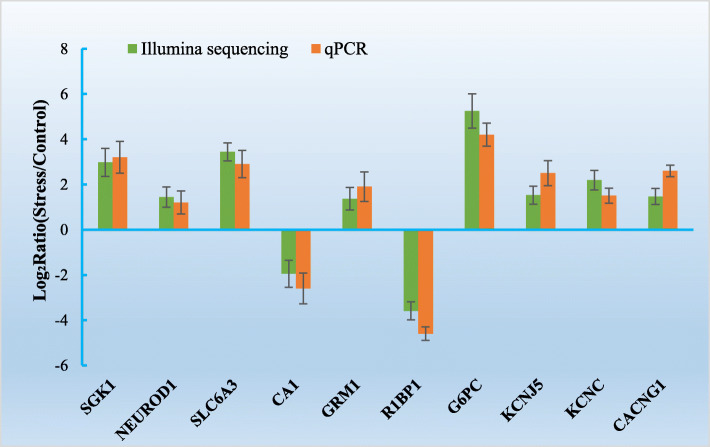
Validation of RNA-seq data. Validation of RNA-seq data was made by qPCR. X-axis, detected gene names; Y-axis, the relative expression level was expressed as log_2_(fold change) in gene expression

**Fig. 4:**
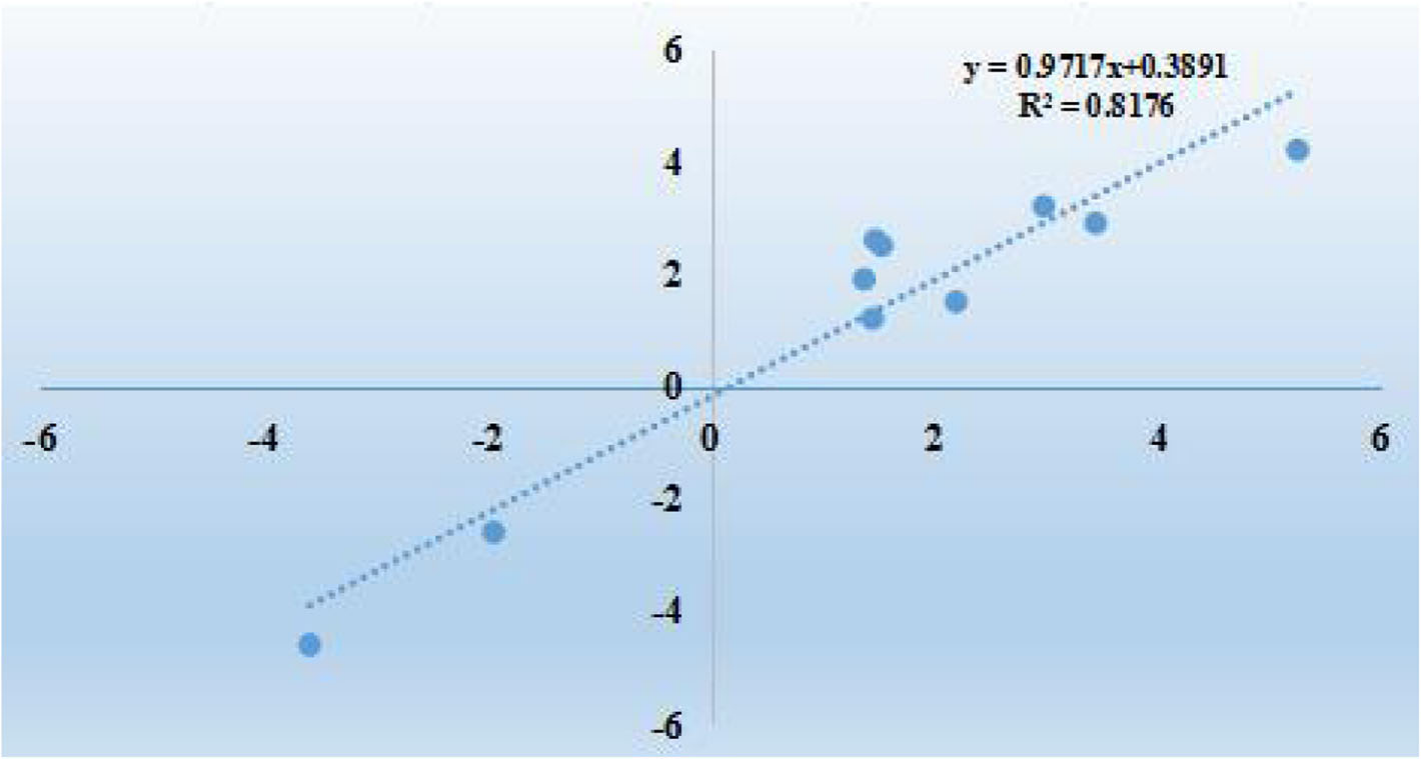
Correlation analysis on the detected ten DEGs

Ten DEGs were randomly selected from the RNA-Seq data of upregulated and downregulated genes. As shown in Fig. 3, expression of the genes were normalized to beta-actin. The genes and primers used for qRT-PCR were shown in Additional file 4: Table S3. *P* values for genes in qRT-PCR were as follows, 0.36, 0.41, 0.25, 0.16, 0.33, 0.43, 0.36, 0.28, 0.18, 0.21. The correlation analysis results for these detected DEGs in the brain are as follows: y = 0.9717x + 0.3891, and R^2^ = 0.8176 (*r* = 0.904, *p* = 0) (Fig. 4), These ten DEGs exhibited a concordant direction in both the RNA-Seq and qPCR analyses.

The results indicated that key pathways and DEGs were mainly involved in the neuronal signal transduction, sensory system, and environmental adaptation. The retrograde endocannabinoid signaling pathway comprehensively regulated glutamate signaling and serotonergic signaling. The DEGs on neuronal signaling pathways involved hormone, neurotransmitter, receptor, transporter, and regulatory protein. For the sensory system, olfactory system signaling pathways were upregulated and phototransduction pathways were downregulated during the saline and cold effect. For the stress response, DEGs relevant to growth inhibition, osmoregulation, and the cold stress response were upregulated.

## Discussion

In this study, we performed a comparative brain transcriptome analysis of *C. nasus* exposed to salinity and cold stress simultaneously. A comprehensive analysis of the top 10 GO and top 10 KEGG terms indicated that the most significantly enriched pathways were involved in three categories: neuronal signaling (neuroactive ligand–receptor interaction, the calcium signaling pathway, the glutamatergic synapse, retrograde endocannabinoid signaling, and the serotonergic synapse), the sensory system (olfactory transduction and phototransduction), and environmental adaptation (ECM–receptor interaction and circadian entrainment). Neurotransmitters, receptors, and key regulatory proteins were shown to be upregulated, constructing a regulatory network in the *C. nasus* brain that plays a role in *C. nasus* migration.

### Quality of the de-novo assembled transcriptome

As shown in Table 1, the ratio of clean reads reached 99.8%, and the reported clean read ratio of high-quality de-novo assembly was around 96% [25, 26]. To some extent, this indicated that we obtained a high-quality assembled dataset in this study. Due to the fact that the reads to be assembled in this study were from different individuals, this added complications, considering that Trinity has a self-correction ability [27]. We also took some actions to refine our dataset, such as filtering the longest open reading frames for each transcript, clustering similar transcripts, and making annotations on filtered transcripts via comparing them with closely related species. Therefore, the assembly result in this study is credible.

### Neuronal signal transduction in *C. nasus* brains under salinity and cold effect

Glutamate is a major excitatory neurotransmitter of the central nervous system. It mediates rapid interneuronal communication and comparatively slow synaptic plasticity, whilst also regulating the ionotropic glutamate receptor (iGluR) and the metabotropic glutamate receptor (mGluR) [28]. In this study, salinity triggered the upregulated expression of glutamate dehydrogenase in the brain, which may be the reason for the upregulation of the glutamate receptor [13]. On the contrary, serotonin exerted a regulatory role in inhibitory neuronal signal transduction and maintained the excitability balance, together with glutamate [29]. The retrograde endocannabinoid signaling pathway was also significantly upregulated. Retrograde endocannabinoid signaling, which plays a homeostatic role in the balance of neuronal activity between the glutamate and inhibitory serotonergic neurotransmitters, is mediated by retrograde messengers, including anandamide and 2-arachidonoyl glycerol (2-AG). These signaling pathways have pivotal regulatory functions in synaptic plasticity, learning, and memory in response to environmental stress [30–32].

In addition to the above-mentioned pathways, many other neurotransmitters, receptors and regulatory proteins were upregulated, such as, galanin, neuropeptide K (NPK), neurotensin (NT), nociceptin and its corresponding receptors, cerebellin-1 (CBLN1), leucine-rich repeat transmembrane neuronal protein 4 (LRRTM4), regulating synaptic membrane exocytosis protein 1 (RIMS), signal-induced proliferation-associated 1-like protein 1 (SIPA1L1), Synaptosomal-associated protein 25 (SNAP25), Teneurin-1 (TENM1), etc. (Table 2). Of these compounds, there is evidence that galanin is involved in the regulation of coping with stressful events [33]. Neurotensin, a key neurotransmitter, plays a regulatory role in easing pain in the brain, for example, by regulating the aversive memory of temperature nociception. Nociceptin and its receptor also play roles in numerous brain activities, such as pain sensation and fear learning [34].

CBLN1, essential for synapse integrity and synaptic plasticity, plays a regulatory role in maintenance of excitatory synapses [35]. LRRTM4, it plays a pivotal role in the development and maintenance of nervous system of vertebrate [36]. RIMS, it plays a regulatory role in neurotransmitter release at the active zone during short-term synaptic plasticity and plays an important role in maintaining the normal probability of neurotransmitter release [37]. SIPA1L1, it functions in promoting reorganization of the actin cytoskeleton and plays a regulatory role in dendritic spine morphogenesis [38]. SNAP25, it plays a key regulatory role in synaptic function of specific neuronal systems, and associates with proteins involved in vesicle membrane fusion [39]. TENM1, as a signal transducer, it plays a regulatory role in the establishment of proper connectivity in the nervous system [40].

### Regulation of the sensory system of *C. nasus* under salinity and cold effect

Salinity is a significant environmental factor during fish migration that can affect the expression of the olfactory receptors, which could be involved in olfactory imprinting. Particularly, for anadromous fish, the sensory system plays a key role in migration [17]. Several studies on the migration mechanism of Salmonids (sockeye salmon, Atlantic salmon, etc.) have concluded that the olfactory epithelium is essential for imprinting and subsequent homing migration as fish return to their natal streams and undergo rapid salinity changes [41, 42]. Under salinity and cold stress condition, as a result, olfactory transduction pathway of juvenile *C. nasus* was significantly upregulated and many relevant regulatory genes were significantly enriched including cyclic nucleotide-gated olfactory channel, claudin, anoctamin, guanylyl cyclase-activating protein, guanylyl cyclase GC-E, etc. (Table 2). Further, the olfactory epithelium associated genes of juvenile *C. nasus* were also shown to be significantly elevated during salinity and cold stress. In contrast, in the Xu et al. [19] study on the Yellow drum (*Nibea albiflora*), the pathways involved in the sensory system were not upregulated, possibly because Yellow drum (*Nibea albiflora*) is a non-anadromous fish species. In *C. nasus*, as an anadromous fish, the sensory system plays an important role in guiding its returning behavior during migration, so its regulatory mechanisms are different from other non-anadromous fish.

Meanwhile, many visual system-relevant genes of juvenile *C. nasus* were shown to be significantly down-regulated during stress exposure, such as rod cGMP-specific 3’,5’-cyclic phosphodiesterase subunit beta, retinol dehydrogenase 7, ventral anterior homeobox, and rhodopsin. Rod cGMP-specific 3’,5’-cyclic phosphodiesterase, a protein involved in the transmission and amplification of visual signals, plays a vital role in the formation of a functional phosphodiesterase holoenzyme. The ventral anterior homeobox is a transcription factor involved in retinal ganglion cell axon guidance. Rhodopsin, a light sensitive G-protein-coupled receptor protein, enables vision in dark conditions [43]. Hale et al. [20] also carried out comparative transcriptome on brain in juvenile resident and migratory smolt rainbow trout (*Oncorhynchus mykiss*) also found that phototransduction signaling pathway and key regulatory genes were expressed significantly. The light-brain-pituitary (LBP) axis transmits information about environmental photoperiod to endocrine systems, the subsequent hormone actions regulate physiological behaviour such as parr-smolt tansformation, etc. [44].

### Response regulation under *salinity and cold effect*

Somatostatin is a peptide hormone that inhibits growth via inhibiting the release of secondary hormones [45]. Cortistatin is a neuropeptide with a high structural similarity and similar function to somatostatin. Furthermore, cortistatin can bind with all somatostatin receptors to mediate its inhibitory effect on downstream growth-associated signaling pathways [46]. In this study, the somatostatin receptor was upregulated in the experimental group to perform an inhibitory role in growth, which is consistent with reports showing that high salinity stress inhibits the growth of tilapia fish [47]. This may be because fish obtain a better energy distribution in response to stress via growth suppression. Heat shock protein 70 (HSP70), a main molecular chaperone, combines with unfolded or misfolded proteins and also promotes the degradation and removal of denatured proteins [48].

In this study, HSP70 was significantly upregulated, and many studies have indicated an elevation of HSP70 in response to environmental stress [49, 50]. HSP70 is also regarded as having the function of inhibiting growth, as well as playing a synergistic role with somatostatin and cortistatin. Research on the regulation mechanism of the brain during salmonid and steelhead trout smoltification showed the differential expression of growth hormone and thyroid hormone receptors [20, 22]. This study also showed the significant differential expression of hormones and receptors related to growth regulation, including cortistatin and the somatostatin receptor, which indicated the importance of growth regulation during anadromous fish migration. Haptoglobin, a stress-activated acid glycoprotein, can bind to free plasma hemoglobin and present itself to the mononuclear macrophage system for processing, in order to maintain homeostasis of the blood micro-environment under stressful conditions [51]. In this study, the haptoglobin concentration increased significantly after stress. A comparative transcriptome analysis of the brain of Atlantic salmon during smoltification showed a significant upregulation of hemoglobin [21]. In this study, hemoglobin was not upregulated, but haptoglobin, which has a regulatory function, was upregulated. Given its binding and presenting function on hemoglobin, this may indicate that Atlantic salmon and *C. nasus* have a similar regulatory pattern in maintaining homeostasis of the blood micro-environment during seaward migration.

In this study, many osmoregulatory-related genes were significantly upregulated, such as claudins, the sodium- and chloride-dependent glycine transporter 2, the solute carrier family (SLC), and the aquaporin and prolactin receptors. Sodium- and chloride-dependent glycine transporter 2 is necessary for Na^+^/Cl^–^-dependent neurotransmitter transportation. SLC is a group of membrane transport proteins, predominantly located in the cell membrane, which play roles in osmoregulation. Aquaporins are integral membrane proteins that control the movement of water in and out of cells and function in osmoregulation [52]. Wang et al. [53] reported that Aquaporin I performs an important regulatory function in *Coilia nasus* under conditions of salinity stress. Prolactin has also been reported to have a regulatory function when freshwater fish swim into seawater [54]. In this study, the prolactin receptors were upregulated to assist with the anti-stress response of *C. nasus*. Prolactin is considered to be an important hormone for salmonid and steelhead trout during the smoltification adaptation process. In this study, the significant up-regulation of the prolactin receptor in *C. nasus* also reflected the important regulatory role of this gene during anadromous fish seaward migration.

Cold stress causes damage to fish through the accumulation of free radicals. Methionine is an effective scavenger for free radicals. It is also one of the methyl donors and participates in the synthesis of adrenaline, creatine, choline, carnitine, and DNA methylation. Inadequate methionine will affect the metabolism of fish and thus weaken its cold resistance [55]. In this study, many genes in the methionine and cysteine receptor pathways were significantly elevated, which also indicated the important role that the methionine metabolism of juvenile *C. nasus* plays in response to cold stress.

According to the theory of the phase transition of membrane lipids, it is believed that the content of unsaturated fatty acids in membrane lipids can affect membrane fluidity and, thus, the cold resistance of fish [56]. Beyond that, cholesterol also plays an important role in maintaining the fluidity of biological membranes [57]. Sterol, a cholesterol-like substance, is widely regarded as a key functional substance in the response to environmental stresses such as extreme temperature and high salinity stress [58, 59]. Sterol 14-demethylase is a key synthetase in the synthesis of cholesterol and phytosterol [60]. In the present study, its expression level was significantly elevated after stress, indicating that the cholesterol level was elevated to enhance the anti-cold resistance of juvenile *C. nasus*.

## Conclusions

In this study, we discovered that neuronal signal transduction played an important regulatory role in maintaining excitability homeostasis. Additionally, the function of the olfactory system was strengthened and the visual signaling pathways were weakened during juvenile *C. nasus* seaward migration. The identified significant differential expression of the growth hormone and receptors; the upregulation of the genes which function in maintaining homeostasis of the blood environment, such as haptoglobin and the prolactin receptors, are similar results to those obtained in research on salmonids and steelhead trout smoltification adaptation. This study revealed an early regulation pattern during juvenile *C. nasus* seaward migration as well as some potential universal regulatory genes which are important during anadromous fish migration. However, this study is more beneficial in terms of revealing the regulation mechanism during juvenile *C. nasus* early seaward migration, and provides a glimpse of the regulation mechanism during *C. nasus* seaward migration. At present, there has not been much research on the regulation mechanism of juvenile *C. nasus* seaward migration, so the pathways and genes identified in this study represent a starting point for further research, and do not represent an exhaustive list of all important candidate genes. Therefore, the regulation mechanism of juvenile *C. nasus* during seaward migration requires more in-depth studies.

## Methods

### Experimental fish and synergistic stress of salinity and low temperature

Experimental fish were five-month-old juvenile *C. nasus* (sex haven’t been distinguished) whose average body length was 133.5 ± 0.65 mm and body weight was 9.66 ± 0.93 g. The fish were collected from Yixing, an experimental base of the Freshwater Fisheries Research Center of the Chinese Academy of Fishery Sciences, in November. Wild juvenile *C. nasus* always migrate to the ocean during this time. One month before the experiment, juvenile *C. nasus* were netted and randomly transferred to six aquariums with circulating water systems whose volume was 1096 × 470 × 670 mm^3^. Each aquarium contained six fish. The aquariums were continuously aerated and the water quality was monitored daily: the water temperature was 20 ± 0.95 °C, the pH was 7.2, and the concentration of dissolved oxygen was 7.9 ± 0.63 mg/L. The experimental fish were fed with compound feed three times each day, at 7:00 a.m.,12:00 p.m., and 6:00 p.m. Feeding stopped 1 day before the stress experiment.

**Fig. 5:**
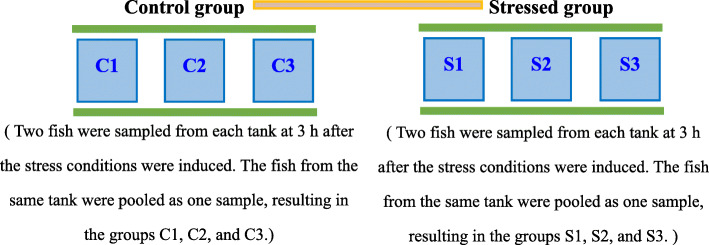
Diagram of the sampling method. C1-C3 indicated three replicated groups in the control group, S1-S3 indicated three replicated groups in the stressed group

The control group (C) and the stressed group (S) were established. The stressed group conditions included a salinity level of 15‰, achieved by adding sodium chloride, and a temperature of 15 °C, achieved by adding crushed ice. Salinity was monitored by using a SALT6 salimeter (Eutech/Oakton, Illinois, USA). The water temperature was monitored by using a RHXL3SD thermometer (OMEGA, Elgin, USA). As shown in Fig. 5, each group had three replicates. It was observed that juvenile *C. nasus* swam gently after 3 h stress treatment., showing an obvious difference in behavior from the initial stress period. Therefore, we sampled at 3 h to discuss internal regulation and adaptation mechanism of juvenile *C. nasus* after stress. Two fish were sampled from each aquarium at 3 h after the stress conditions were induced. A barrel containing 40 mg/L MS-222 (Kuer Bioengineering, Beijing, China) was prepared in advance. Fish were netted quickly and placed in it for 10 s for immersion anaesthesia at the time of collection at 10 am. We cut an incision from fish’s neck for bloodletting to sacrifice fish, then we cut the skull above brain from the incision with scissors, took out the brain with forceps, put it in liquid nitrogen for snap-frozen, and then stored at -80 °C.

### Total RNA extraction, cDNA library construction, and Illumina sequencing

Total RNA was extracted from each brain sample using RNAiso reagent (Takara, Kusatsu-Shiga, Japan), in accordance with the manufacturer’s instructions. Then, equal amounts of total RNA of individuals from each aquarium were pooled. Finally, six RNA brain samples were obtained (Fig. 5), and the amount of RNA of each sample was 1 μg. The concentration and quality of extracted RNA were checked on an Agilent Bioanalyzer 2100 with RNA 6000 Nano Labchips (Agilent technologies, Santa Clara, USA), and contaminant genomic DNA was removed with Recombinant DNaseI (Takara, Kusatsu-Shiga, Japan). The mRNA was isolated with magnetic beads and then broken into fragments and reverse transcribed into cDNA with added adapters. Finally, six cDNA libraries were constructed and sequenced on the Illumina HiSeq™ 2500 platform (Illumina, San Diego, USA). Paired-end data were used for further analysis. The obtained raw data were submitted to NCBI (NCBI, Bethesda, USA) with the accession number SRP078492.

### Data filtering and assembly

The raw data were tested for quality control using FASTQC (Babraham Institute, Cambridge, UK). Some low-quality vectors (including adapters and/or primers), contaminated reads, low-quality bases at the 3’ end, empty reads, and ambiguous ‘N’ nucleotides were removed, and the cutoff value for length control was set as 35 bp. NGS QC TOOLKIT v2.3.3 (Roche, Pleasanton, USA) (http://59.163.192.90:8080/ngsqctoolkit/) [61] was used to filter the above-mentioned data. Transcriptome assembly was performed using the Trinity software (Broad institute, Cambridge, UK) [27].

### Function annotation, gene quantification, and differential expression analysis

The similarity alignment was based on the BLAST algorithm (NCBI, Bethesda, USA). For homology annotation, the obtained non-redundant sequences were aligned in the following priority order: non-redundant protein (Nr), non-redundant nucleotides (Nt), Swiss-prot (http://www.uniprot.org/downloads), clusters of orthologous groups for eukaryotic complete genomes (KOG, ftp://ftp.ncbi.nih.gov/pub/COG/KOG/kyva), gene ontology (GO, http://www.geneontology.org/), and the Kyoto Encyclopedia of Genes and Genomes.

(KEGG, http://www.genome.jp/kegg/pathway.html) [62, 63]. The unigenes [64] were searched in public databases, including Nr, Nt, Swiss-prot, and KOG, with an E-value cutoff of 10^–5^. GO annotation of the obtained unigenes was performed using Blast2GO software (Biobam, Valencia, Spain) [65].

The assembled unigenes were placed in a constructed library, and the expression of the abundance of each unigene in each sample was measured by bowtie2 software (http://bowtie-bio.sourceforge.net/bowtie2/manual.shtml) (Ben Langmead, Maryland, College Park, USA) [66] and eXpress software (http://www.rna-seqblog.com/express-a-tool-for-quantification-of-rna-seq-data/) (California university, Berkely, USA) [67]. FPKM was used to evaluate the gene expression levels.

Differential expression quantification was calculated using the DESeq software package (http://bioconductor.org/packages/release/bioc/html/DESeq.html) [68]. Fold change was calculated as the ratio of the expression level of genes in the stressed group and control group samples, and |log_2_foldchange| ≥ 1 and FDR < 0.05 (Benjaminie–Hochberg false discovery rate) [69] were set as the cutoff thresholds to determine the significantly differentially expressed genes. GO and KEGG pathway enrichment analyses were performed on differentially expressed genes (FDR < 0.05). The GO and KEGG terms were sequences with –log10 (*p*-value), and terms with a list hit of less than two were excluded (list hit refers to number of DEGs enriched in one GO or KEGG term). Finally, we obtained the top 10 GO terms and top 10 KEGG pathways in the brain. According to the pathway hierarchy that the KEGG website published (Additional file 3: Table S2), the top 10 KEGG pathways were further categorized. Considering the top 10 GO terms, the top 10 KEGG pathways, key pathways, and DEGs relevant to the anti-stress response, a regulatory network was constructed.

### Quantitative real-time PCR validation

A quantitative real-time polymerase chain reaction (qPCR) was performed with the extracted RNA to validate the accuracy of the transcriptome sequencing analysis by the ABI 7500 Real-Time PCR System (ABI, New York, USA) using the Power SYBR™ Green PCR Master Mix. Ten differentially expressed genes were randomly selected with the Randbetween Function and the primers were designed with Primer Premier 5 software (Biosoft, California, USA). β-actin was used as the internal reference. The amplifications were conducted with the following procedure: 95 °C for 30 s, 40 cycles of 95 °C for 5 s, 60 °C for 34 s, and 72 °C for 50 s. Every sample was analyzed in triplicate, and the permitted variation of the cycle threshold in replicates was 8%. The 2^-ΔΔCT^ method was used to calculate the gene expression level [70].

### Statistical analysis

The statistical analysis for qPCR experimental data was completed with SPSS 21.1 software (SPSS, Chicago, USA), and values are shown as means ± SD. All data were subjected to one-way ANOVA and Tukey’s multiple range tests. The α-level was set at 0.05.

## Supplementary information

**Additional file 1 **Appendix file 1: **Figure S1**: GO classification of unigenes.

**Additional file 2 **Appendix file 2: **Table S1:** top 10 GO terms, top 10 KEGG pathways and all DEGs in this study.

**Additional file 3 **Appendix file 3: **Table S2**: pathway hierarchy.

**Additional file 4 **Appendix file 4: **Table S3**: The genes and primers used for real-time RT-PCR validation.

**Additional file 5 **Appendix file 5: **Table S4**: FPKM of DEGs in the control and stressed group.

## Data Availability

The dataset supporting the conclusions of this article is available in the NCBI Sequence Read Archive (SRA) repository, accession number SRP078492. (https://www.ncbi.nlm.nih.gov/sra/SRP078492). Supplementary data to this article can be found online at 10.17632/p2k764sxf3.1 and 10.17632/nhnswvxf7w.1.

## Abbreviations

*C. nasus*: *Coilia nasus*
DEGs: Differentially expressed genes
qPCR: quantitative real time polymerase chain reaction
RIN: RNA integrity number
FPKM: Fragments per kilobase of transcript per million mapped reads
ECM: Extracellular matrix
GO: Gene Ontology
BP: Biological process
MF: Molecular function
CC: Cellular component
KEGG: Kyoto Encyclopedia of Genes and Genomes
iGluR: Glutamate receptor ionotropic
mGluR: Metabolic glutamate receptor
2-AG: 2-arachidonoyl glycerol
GABR: Gamma-aminobutyric acid receptor
CNR1: Cannabinoid receptor
NPY: Neuropeptide Y
NPK: Nerupeptide K
NT: Neurotensin
CBLN1: Cerebellin-1
LRRTM4: Leucine-rich repeat transmembrane neuronal protein 4
RIMS: Regulating synaptic membrane exocytosis protein 1
SIPA1L1: Signal-induced proliferation-associated 1-like protein 1
SNAP25: Synaptosomal-associated protein 25
TENM1: Teneurin-1
SLC: Solute carrier family
HSP70: Heat shock protein 70
Nr: Non-redundant protein
Nt: Non-redundant nucleotides
KOG: Clusters of orthologous groups for eukaryotic complete genomes.
