# Supplementary figures and images for: Transcriptome analysis of the brain provides insights into the regulatory mechanism for Coilia nasus migration
Source: BMC Genomics. 2020 Jun 18;21:410. doi: 10.1186/s12864-020-06816-3 (PMC7302372; doi:10.1186/s12864-020-06816-3)

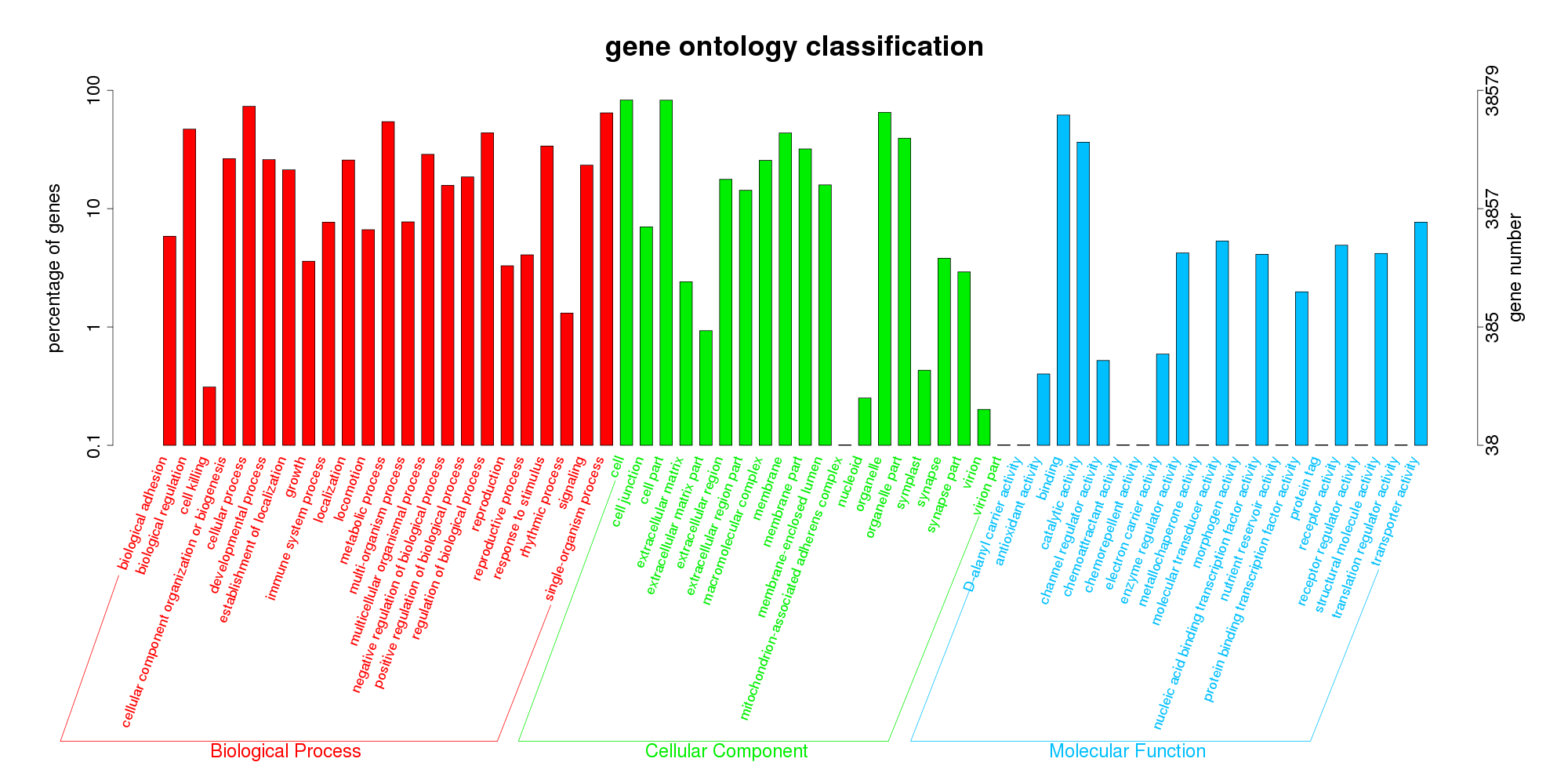


Figure S1. GO classifications of unigens.

Supplement: Supplementary file 1 — Additional file 1 Appendix file 1: Figure S1: GO classification of unigenes. [file 12864_2020_6816_MOESM1_ESM.docx]
