# Supplementary material for: Transcriptome analysis of the brain provides insights into the regulatory mechanism for Coilia nasus migration
Source: BMC Genomics. 2020 Jun 18;21:410. doi: 10.1186/s12864-020-06816-3 (PMC7302372; doi:10.1186/s12864-020-06816-3)
Supplement: Supplementary file 4 — Additional file 4 Appendix file 4: Table S3: The genes and primers used for real-time RT-PCR validation. [file 12864_2020_6816_MOESM4_ESM.docx]

**Table S3. The genes and primers used for Real-time RT-PCR validation**

| Number | Gene name | Gene definition | Sequence(5’→3’) |
| --- | --- | --- | --- |
| 1 | SGK1 | Serum/glucocorticoid-regulated kinase 1 | F: GCCTCCATTCTACAGTCGC |
|  |  |  | R: CGTCCCAGTTGATGGGAGAG |
| 2 | NEUROD1 | neurogenic differentiation factor 1 | F: CCACAAATCTAGTGGCGGG |
|  |  |  | R: CATACGCGTGTGGTTTGACG |
| 3 | SLC6A3 | sodium-neurotransmitter symporter | F: CAAGGATTCGGCTCCTACCG |
|  |  |  | R: GCCAGGAAGAACAGACGTG |
| 4 | CA14 | carbonic anhydrase 14 | F: GTCAGTCCCCCATCAATGTC |
|  |  |  | R: CTATGGCTGCGATTTCACC |
| 5 | GRM1 | metabotropic glutamate receptor 1 | F: GCTACATGTACGACAACGCC |
|  |  |  | R: GACGCGTAAGTCATGCTTGG |
| 6 | RLBP1 | retinaldehyde-binding protein 1 | F: GGCTTCTGCCTGATTGAG |
|  |  |  | R: GGTCAGGAGGTTAAAGGGC |
| 7 | G6PC | glucose-6-phosphatase | F: GCTCATATGGGTCGCTGTG |
|  |  |  | R: CAGACCCCATTGCATGACC |
| 8 | KCNJ5 | potassium inwardly-rectifying channel subfamily J member 5 | F: GGTATCTGGCATGCTGTTGG |
|  |  |  | R: GATGTCACATTGTGCTGGC |
| 9 | KCNC | voltage-gated K^+^ channel | F: CAGCATGGAACTCATGGACG |
|  |  |  | R: CGGCACAAACTCATAGAGGC |
| 10 | CACNG1 | voltage-dependent calcium channel gamma-1 | F: CTACTTACTGAGGCCGTCG |
|  |  |  | R: CTCCATCCAAACGGTCTCC |
| 11 | β-actin | beta-actin | F: GCAACACGCAGCTCGTTGTAG |
|  |  |  | R: CAGGCATCAGGGTGTGATGG |
